# Posttraumatic growth and resilience among survivors of cancer in young adulthood in Switzerland: a report from the Adolescent and Young Adult (AYA) Psychosocial Health Study

**DOI:** 10.1007/s00520-026-11383-6

**Published:** 2026-11-04

**Authors:** Céline Bolliger, Luzius Mader, Mohsen Mousavi, Marcel Blum, Beat Müller, Marcus Vetter, Katharina Roser

**Affiliations:** 1 Faculty of Health Sciences and Medicine, University of Lucerne, Alpenquai 4, 6005 Lucerne, Switzerland; 2 Cancer Registry Bern and Solothurn, Bern, Switzerland; 3 Cancer Registry of Eastern Switzerland, St. Gallen, Switzerland; 4 Department for Medical Oncology, Cantonal Hospital of Lucerne, Lucerne, Switzerland; 5 Center for Oncology and Hematology, Cancer Center Baselland, Cantonal Hospital Baselland, Liestal, Switzerland

**Keywords:** Young adult cancer, Survivorship, Posttraumatic growth, Resilience

## Abstract

**Purpose:**

Survivors of young adult cancer (YACS) face distinct psychosocial challenges. Posttraumatic growth (PTG) and resilience may aid adaptation, though their relationship and relevance in this age group remain insufficiently understood. We aimed to (1) describe PTG and resilience among Swiss YACS and (2) investigate the associations of PTG with resilience, sociodemographic, and cancer-related characteristics.

**Methods:**

We included survivors aged 21–39 years at diagnosis, diagnosed at least two years prior. We conducted a questionnaire survey using the Posttraumatic Growth Inventory (PTGI; range:0–105) and the Connor-Davidson Resilience Scale (CD-RISC 10; range:0–40). Data were analyzed using descriptive statistics and linear regression.

**Results:**

A total of 131 YACS (62% female; mean age at study = 37.6 years, SD = 5.1) were included. The PTG sum score was 54.6 (SD = 23.0, range:1–101), and YACS reported most PTG in *Appreciation of life* (mean = 3.3, 95% confidence interval: 3.2–3.6) and least in *Spiritual change* (1.1, 0.9–1.3). The mean resilience score was 28.06 (SD = 5.91, range:10–39), and YACS reported the highest score for the item “Achieving goals despite obstacles” (3.2, 2.6–3.0), and the lowest for the item “See humorous side of things” (2.1, 2.0–2.3). PTG was higher among females and those who received chemotherapy, while resilience showed no significant association with PTG.

**Conclusion:**

These findings suggest that PTG and resilience are distinct but complementary adaptation processes in YACS. With higher PTG linked to female gender and chemotherapy, and resilience showing no direct association with PTG, personalized psychosocial support remains essential to help survivors navigate their unique cancer experiences and promote well-being.

**Supplementary Information:**

The online version contains supplementary material available at https://doi.org/10.1007/s00520-026-11383-6.

## Background

Cancer in *adolescents and young adults* (AYAs; 15–39 years of age, although its upper boundary varies) differs from cancer in children or older adults in terms of tumor biology, distribution of cancer types, psychosocial needs, and late outcomes following treatment [1–4]. Thanks to better treatments, the 5-year survival rate increased to about 80% for AYAs diagnosed with cancer in Europe [5, 6]. As survival rates improve, the number of AYA cancer survivors continues to grow, and it has become increasingly recognized that they represent a distinct group requiring targeted efforts to improve their lives during and after treatment [7, 8]. These efforts are crucial, as many survivors continue to experience enduring physical and psychosocial consequences (so-called late effects) of their cancer diagnosis and treatment [9, 10]. As a result, many survivors might experience negative consequences, including a higher risk of cardiovascular diseases, endocrine disorders and mental health disorders, social isolation, and significant disruptions in education and career planning [9, 11–13].

Despite all the negative consequences of surviving cancer, literature has shown that dealing with a potentially traumatic event, such as getting diagnosed with cancer [14], might have a silver lining: people might “benefit” from the experience and report growth, such as more appreciation for life or intensified personal relationships [15–17]. Posttraumatic growth (PTG) describes a salutogenetic concept and a phenomenon in which individuals experience changes that surpass their pre-existing state after facing a burdensome experience [18]. In fact, it leads to growth and meaning-making in five domains of a person’s life: relating to others, new possibilities, personal strength, spiritual change, and appreciation of life [19]. A previous study in Swiss AYA cancer survivors aged 16 to 25 years at diagnosis has shown that all of the participants reported at least some PTG in the aftermath of cancer [20]. However, it did not cover the whole AYA age range up to 39 years.

Another salutogenic concept is resilience, describing the phenomenon of the level of adaptation when facing adversity, trauma, tragedy, threats, or significant sources of stress [21]. In contrast, posttraumatic growth does not refer to maintaining functioning during adversity, but to positive psychological changes that arise from struggling with traumatic experiences and life disruption [22]. It is debated as to whether or not PTG is a form of resilience, and whether or not growth is superior to resilience [23, 24]. A recent study described resilience as a relatively stable personality trait, rooted in dispositions such as optimism, emotional stability, and self-efficacy, which helps individuals cope with both everyday stress and traumatic experiences [25].In the present study, we follow the conceptualization of resilience as a stress-coping capacity that can be strengthened over time, as originally proposed by Connor and Davidson [26], which underlies both our use of the CD-RISC 10 and the interpretation of our findings.

Despite the often presented positive relationship between PTG and resilience in cancer patients and survivors, the strength of this association appears to vary across studies [27–30]. A recent study with AYA cancer survivors found a strong positive correlation between PTG and resilience, indicating that higher resilience was associated with higher levels of PTG in this population [30]. Similarly, Gündoğmuş et al. (2022) observed a positive relationship between resilience and PTG in breast cancer patients, emphasizing the role of resilience in facilitating coping and adjustment [28]. In another study, also with breast cancer patients, PTG was again positively associated with resilience, although it explained only 4% of the variance in the resilience scores, pointing to a weaker connection [29].

Although research on PTG and resilience in AYA cancer survivors remains limited, these constructs may play a key role in supporting their adjustment after cancer. This gap in age at diagnosis coverage, combined with inconsistent evidence on the PTG–resilience relationship in cancer survivors more broadly [27–30], underscores the need to clarify these constructs' interplay in a wider AYA cancer sample to inform differentiated, age-tailored psychosocial support. Understanding and fostering PTG and resilience in AYA cancer survivors is crucial to improving their long-term quality of life and ensuring they receive appropriate care if in need.

The proposed study aims to (1) describe PTG and resilience among Swiss survivors of young adult cancer (YACS) and (2) investigate the associations between PTG, sociodemographic and cancer-related characteristics, and resilience.

## Methods

### Sample and procedure

Study participants were recruited from hospitals in the German-speaking part of Switzerland. The clinics were identified with the support of Swiss cancer registries, from which we received lists of doctors who treat AYAs with cancer in Switzerland. These doctors were contacted and asked to collaborate by identifying former patients (YACS) and inviting them to participate in the current study. Survivors aged 21–39 at diagnosis were eligible, as survivors aged 15–20 at diagnosis were already covered by the Swiss Childhood Cancer Survivor Study (SCCSSS) [31]. They were German-speaking and were treated in Switzerland and diagnosed between 2010 and 2019, and survived for at least two years after their diagnosis. Between February 2023 and June 2024, AYA cancer survivors were sent a study package, including the cover letter, the study information, a consent form, the questionnaire, as well as a link for the online questionnaire, and a pre-paid return envelope. After 6 weeks, a reminder letter was sent to those who had not responded. All procedures performed in this study were in accordance with the ethical standards of the responsible research committee and with the 1964 Helsinki Declaration and its later amendments or comparable ethical standards. The study was approved by the Ethics Committee Northwest and Central Switzerland (EKNZ 2022–01065, 12 October 2022).

### Measurements

In total, the survey consisted of 185 items and was developed using a combination of standardized and self-developed questions regarding the survivors’ psychosocial health and needs, and their socio-demographic and cancer-related characteristics.

#### Posttraumatic growth

We assessed PTG using the German version of the Posttraumatic Growth Inventory (PTGI), which consists of 21 items and is well-validated [26]. It compromises five domains: *Relating to others* (7 items), *New possibilities* (5 items), *Personal strength* (4 items), *Spiritual change* (2 items), and *Appreciation of life* (3 items). Each item is rated using a 6-point Likert scale (0 = not at all to 5 = extremely). We calculated the mean for each of the five domains (possible range: 0–5) and the PTG sum score as the sum of all item scores (possible range: 0–105), with higher scores indicating higher levels of PTG. Based on previous publications, we categorized the answers into low (0,1), middle (2,3) and high (4,5) [16, 20] for descriptive purposes only while for the regression analyses, we used the continuous PTG sum score. We replaced missing PTG values with the mean of the respective domain if at least half of the items within that domain were available. Participants with insufficient valid responses in a subscale (i.e., less than half of the items) were excluded from that specific subscale analysis. Finally, we calculated the PTG mean score being the mean of all item scores (possible range: 0–5). Internal consistency of the PTGI in the present sample was excellent (Cronbach's α = 0.95).

#### Resilience

Resilience was assessed using the German version of the Connor-Davidson Resilience Scale (CD-RISC 10) consisting of 10 items [32]. It assesses various aspects of resilience, including *Adapt to change*, *Can deal with whatever comes*, *Coping with stress strengthens, See humorous side of things*, *Tend to bounce back after illness or hardship*, *You can achieve your goals, Under pressure, focus and think clearly*, *Not easily discouraged by failure*, *Think of self as strong person, Can handle unpleasant feelings.* Each item is rated using a 5-point Likert scale (0 = not at all true to 4 = almost always true). We categorized the answers into low (0,1), middle (2), and high (3,4) for descriptive purposes only, while the continuous score was used as predictor in the regression analyses. Scores were computed by summing the responses, with missing responses (up to 3 items) imputed using the mean of the completed items; scores with fewer than 7 completed items were considered invalid [33]. The possible range of the total sum score is 0–40, with higher scores reflecting higher levels of resilience. Internal consistency of the CD-RISC 10 in the present sample was good (Cronbach's α = 0.85).

#### Sociodemographic and cancer-related characteristics

Sociodemographic characteristics, including the survivors’ age at study (assessed continuously and categorized into 21–30, 31–39, ≥ 40 years), gender (female, male, diverse), highest educational achievement (compulsory schooling, vocational training, upper secondary education, university degree), employment status (full-time employed, part-time employed, homeworker, unemployed), partnership status (yes, no), and migration background (yes, no; survivors were classified as having a migration background if they were not Swiss citizens, not Swiss citizens since birth or were not born in Switzerland), were assessed in the questionnaire. For further analysis, we categorized the highest educational achievement into low (compulsory schooling, vocational training) and high (upper secondary education, university education), and employment status into employed (full-time, part-time) and other situations (homeworker, unemployed).

We assessed information on diagnosis (classified according to the AYA cancer classification by Barr et al. [34]; leukemias and related disorders, lymphomas, CNS and other intracranial and intraspinal neoplasms, sarcomas, blood and lymphatic vessel tumors, nerve sheath tumors, gonadal and related tumors, melanoma malignant, carcinomas). For further analysis, we categorized information on diagnosis into carcinomas, gonadal tumors, lymphomas, and other tumors (leukemias, CNS neoplasms, sarcomas, nerve sheath tumors, and malignant melanoma). We assessed information on age at diagnosis (assessed continuously and categorized into 21–30, 31–39 years), time since diagnosis (assessed continuously and categorized into 2–5, 6–9, 10–13 years), and treatment (hierarchically coded as surgery only, chemotherapy (may have had surgery), radiotherapy (may have had surgery and/or chemotherapy)), stem cell transplantation (may have had surgery and/or chemotherapy and /or radiotherapy). For further analysis, we categorized treatment hierarchically into surgery (surgery only), chemotherapy (may have had surgery), and radiotherapy and stem cell transplantation (may have had surgery and/or chemotherapy). We asked survivors whether they had experienced any late effects, cancer relapses, or second malignancies (yes, no). For n = 103 survivors, cancer-related data from the corresponding cancer registry were available.

### Statistical analysis

We performed all statistical analyses using Stata 19.0 (StataCorp, TX). First, descriptive statistics were calculated for sociodemographic and cancer-related characteristics to describe the study population. We compared our YACS sample with the corresponding YACS population in Switzerland. Where cancer-related data from the questionnaire was missing (diagnosis: *n* = 2, year of diagnosis: *n* = 4, treatment: *n* = 3, relapse: *n* = 3, second cancer: *n* = 3), information was imputed using the cancer-related data from the cancer registries, where available. Additionally, we checked and validated the cancer-related data from the questionnaire for the survivors where we had data from the registry available and added information from the registry if the answer from the questionnaire was not complete (treatment: *n* = 19; no other disagreements). We used descriptive statistics and calculated chi-square tests for categorical variables to compare our YACS sample with the corresponding YACS population in Switzerland. *P* values < 0.05 were considered statistically significant if not stated otherwise.

For aim 1, we used descriptive statistics to describe PTG and resilience. We had to exclude *n* = 3 participants from the respective analysis as more than half of the items within one domain of the PTGI were missing. Regarding resilience, we replaced missing responses with the mean of the completed items if at least 7 items were completed (*n* = 4) and scores with fewer than 7 completed items were considered invalid (*n* = 3).

For aim 2, we ran univariable and multivariable linear regression analyses to investigate the associations of PTG with resilience, sociodemographic, and cancer-related characteristics. All variables that were statistically significant at *p* < 0.1 in the univariable model were included in the multivariable model. *P*-values for categorical independent variables with more than two levels were calculated using the Wald test. For the multivariable models, normality of residuals and homoscedasticity were assessed and considered adequately met. Additionally, we conducted an explorative analysis of the associations of the resilience items with the PTG sum score which was motivated by the possibility that associations between specific resilience components and PTG might be masked when only the aggregated CD-RISC 10 sum score is considered. We further examined associations of the different characteristics assessed with the five PTGI domains, similarly motivated by the possibility of domain-specific effects masked by the global PTG score.

## Results

### Sample characteristics

We contacted 678 YACS and received 236 responses (34.8%). Of these, 175 (25.8%) were positive responses (e.g., we received either informed consent or the questionnaire), and 131 participants (19.3%) could be included in our analysis (Supplementary Information, Figure S1). The study population and the YACS population of Switzerland were similar regarding gender and age at diagnosis, but not regarding diagnosis, treatment, and second cancer (Supplementary Information, Table S1). The study sample included more gonadal tumors (24% vs. 16%) and fewer lymphomas (18% vs. 11%) and melanomas (8% vs. 14%), and more survivors treated with chemotherapy (33% vs. 16%), radiotherapy (37% vs. 21%), and stem cell transplantation (7% vs. 1%), and more with a second cancer (7% vs. 2%).

Participating survivors had a mean age at study of 37.6 years (SD = 5.1, Table 1), and 81 (61.8%) of them were females. Most survivors had completed upper secondary education (*n* = 53, 40.5%) and were employed full-time (*n* = 56, 42.8%). Main diagnoses were carcinomas (*n* = 52, 39.7%), gonadal tumors (*n* = 31, 23.7%) and lymphomas (*n* = 23, 17.5%).

**Table 1:** Sociodemographic and cancer-related characteristics of study participants

	Survivors of young adult cancer (*n* = 131)			
	*n* (%)	%		
*Sociodemographic characteristics*				
Gender				
Female	81	61.8		
Male	50	38.2		
Age at study				
21–30 years	13	9.9		
31–39 years	71	54.2		
40–44 years	39	29.8		
45–49 years	5	3.8		
≥ 50 years	3	2.3		
Migration background				
No	126	96.2		
Yes	5	3.8		
Educational achievement				
Compulsory schooling	2	1.5		
Vocational training	48	36.6		
Upper secondary education	53	40.5		
University education	28	21.4		
Employment status at study				
Employed full-time	56	42.8		
Employed part-time	34	25.9		
Homeworker	27	20.6		
Not employed	14	10.7		
Partnership at study				
Yes	102	77.9		
No	29	22.1		
*Cancer-related characteristics*				
Diagnosis (Barr et al. 2020)^a^				
Carcinomas	52	39.7		
*Breast cancer*	*33*	*25.2*		
*Colorectal or anal cancer*	*12*	*9.1*		
*Thyroid cancer*	*5*	*3.8*		
*Pancreatic cancer*	*1*	*0.8*		
*Bladder cancer*	*1*	*0.8*		
Gonadal tumors	31	23.7		
Lymphomas	23	17.5		
Other tumors^b^	25	19.1		
Treatment^c^				
Surgery	30	22.9		
Chemotherapy	43	32.8		
Radiotherapy	49	37.4		
Stem cell transplantation	9	6.9		
Age at diagnosis				
21–30 years	50	38.2		
31–39 years	81	61.8		
Time since diagnosis				
2–5 years	56	42.8		
6–9 years	56	42.8		
10–13 years	19	14.4		
Late effects				
No	86	65.7		
Yes	45	34.3		
Relapse				
No	119	90.8		
Yes	12	9.2		
Second cancer				
No	122	93.1		
Yes	9	6.9		
	**Mean**		**SD**	**Range**
Age at study (in years)	37.6		5.12	25–52
Age at diagnosis (in years)	31.3		5.04	21–40
Time since diagnosis (in years)	6.3		2.76	2–13

Abbreviations: *CNS* central nervous system, *n* = number, *SD* standard deviation

^a^ Cancer categories according to the cancer classification by Barr et al. [34]

^b^ The category “other tumors” consists of leukemias, CNS neoplasms, sarcomas, nerve sheath tumors, and malignant melanoma

^c^ Hierarchically coded: chemotherapy may include surgery, radiotherapy may include surgery and/or chemotherapy, stem cell transplantation may include surgery and/or chemotherapy and/or radiotherapy

### Posttraumatic growth

**Fig. 1:**
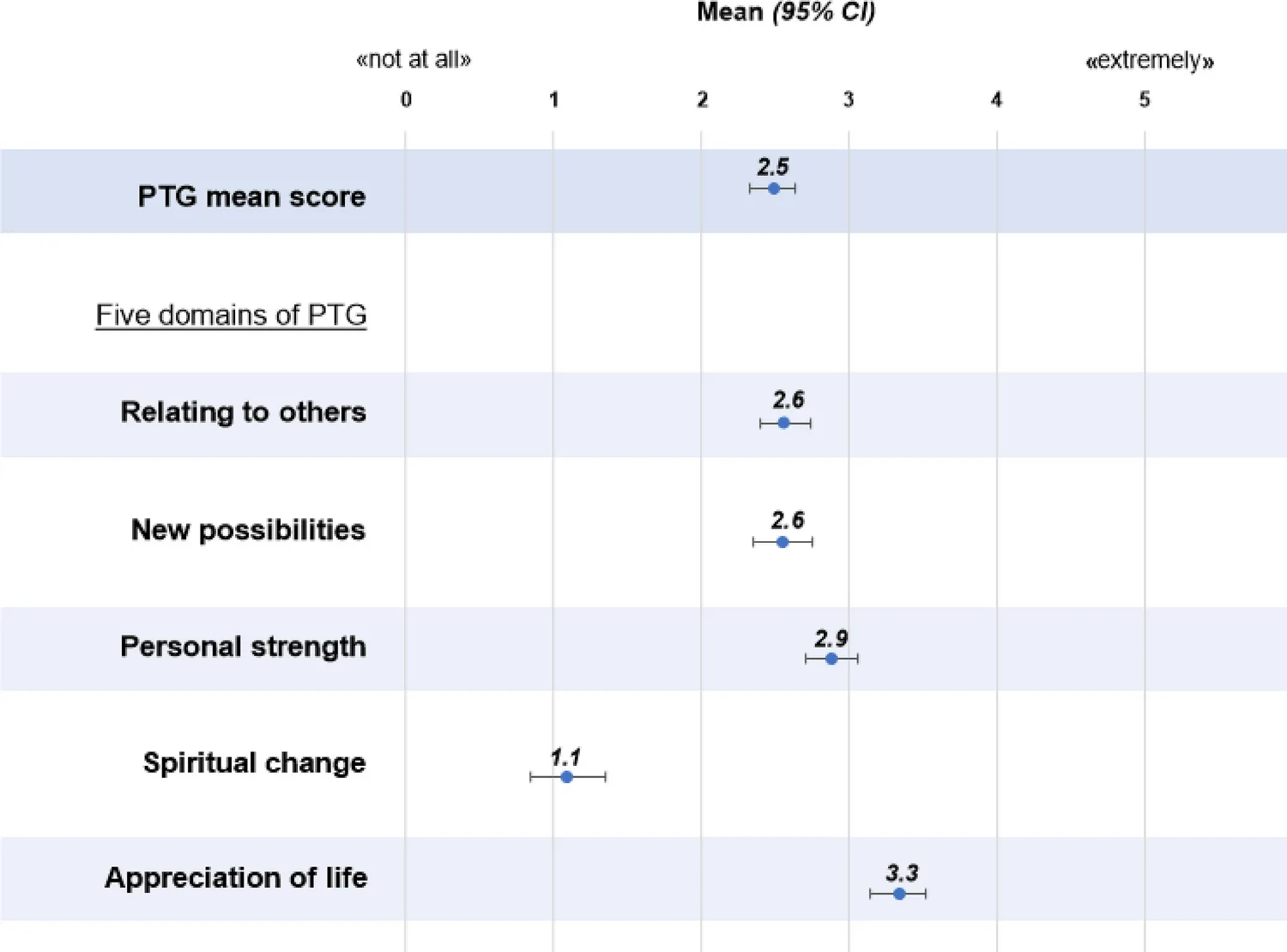
Means and 95% confidence intervals (CI) of the PTG mean score and the PTG domains measured by the PTGI

The mean PTG sum score was 54.6 (SD = 23.0, range: 1–101), and the mean PTG mean score was 2.5 (SD = 1.1, range: 1–5). YACS reported most PTG in *Appreciation of life* and least in *Spiritual change* (Fig. 1).

The four most endorsed items were from *Appreciation of life*: “My priorities about what is important in life” and “An appreciation for the value of my own in life”, from *Relating to others*: “Knowing that I can count on people” and from *Personal strengths*: “Knowing that I can handle difficulties” (Supplementary Information, Figure S2). The three least endorsed items were from *Spiritual change*: “A better understanding of spiritual matters” and “I have stronger religious faith”, and from *New possibilities*: “New opportunities are available which wouldn’t have been otherwise”.

### Resilience

**Fig. 2:**
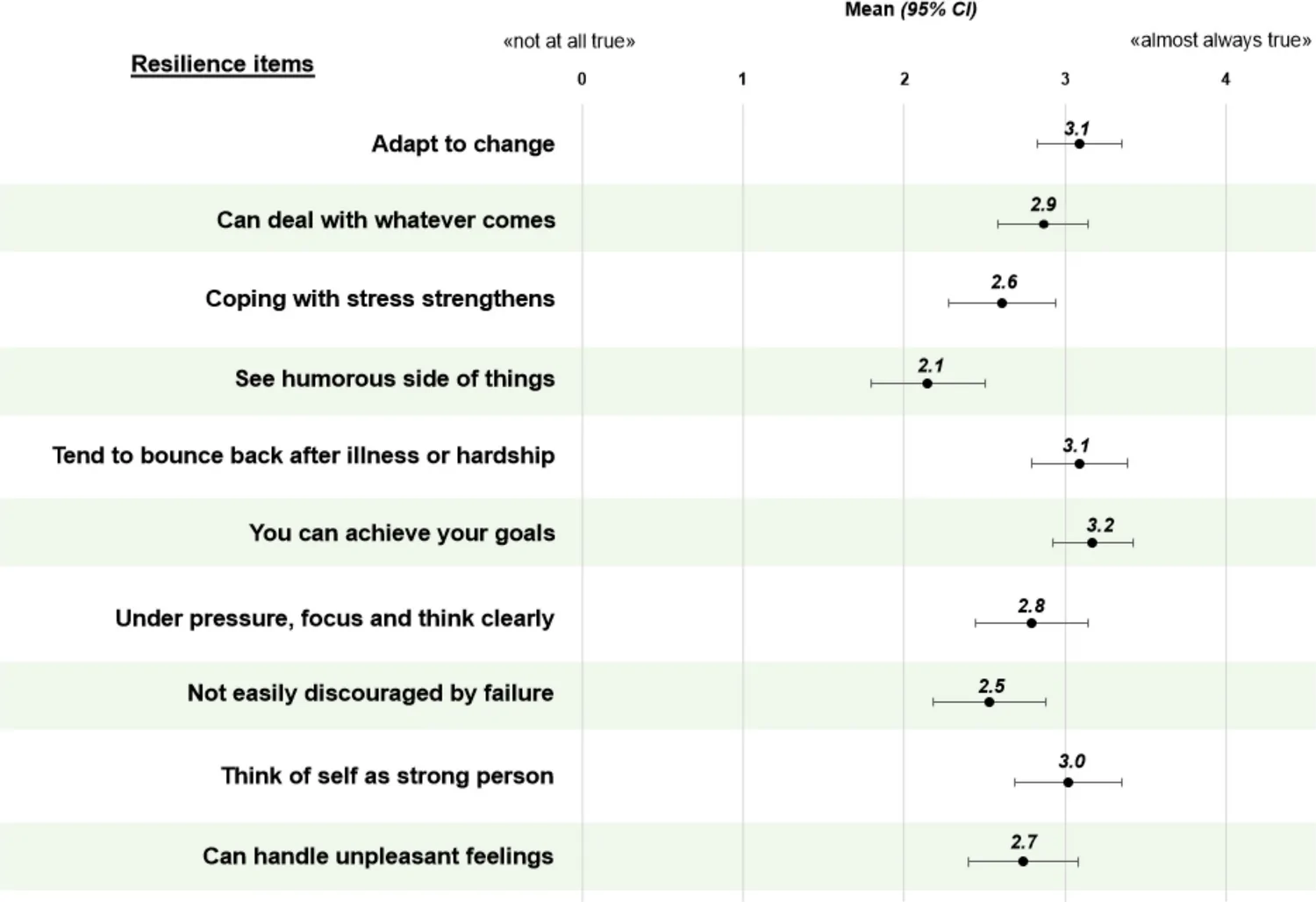
Means and 95% confidence intervals (CI) of the resilience items measured by the CD-RISC 10

The mean resilience score was 28.1 (SD = 5.9, range: 10–39). YACS reported the highest resilience scores within the item “You can achieve your goals” and least within the item “See humorous side of things” (Fig. 2).

The three most endorsed items of the CD-RISC 10 were: “You can achieve your goals”, “Adapt to change”, and “Tend to bounce back after illness or hardship”. The three least endorsed items were: “Coping with stress strengthens”, “Not easily discouraged by failure”, and “See humorous side of things” (Supplementary Information, Figure S3).

### Associations of PTG with sociodemographic and cancer-related characteristics, and resilience

Table 2 shows the univariable and multivariable linear regressions investigating the associations of sociodemographic and cancer-related characteristics and resilience with PTG (PTG sum score). Female sex was significantly associated with higher PTG scores compared to male sex (10.08; *p* = 0.038). Regarding treatment, participants who had received chemotherapy showed significantly higher PTG scores than those who had undergone surgery only (15.62; overall *p* = 0.014).

**Table 2:** Univariable and multivariable linear regressions investigating the associations of sociodemographic and cancer-related characteristics, and resilience with PTG (PTG sum score)

	Univariable linear regression			Multivariable linear regression		
	Coeff	95% CI	*p* value	Coeff	95% CI	*p* value^e^
Sociodemographic characteristics						
Sex			**0.001**			**0.038**
Male	Ref			Ref		
Female	13.59	5.71—21.45		10.08	0.06—19.56	
Age at study			0.705			
21–30 years	Ref					
> = 31	2.16	−11.19—15.51				
Migration background			0.383			
No	Ref					
Yes	−9.19	−29.96—11.59				
Educational achievement^a^			0.945			
Low	Ref					
High	−0.29	−8.51—7.93				
Employment status^b^			0.553			
Employed	Ref					
Other situation	2.62	−6.10—11.34				
Partnership			0.161			
No	−6.80	−16.35—2.74				
Yes	Ref					
Cancer-related characteristics						
Diagnosis			**0.010**			0.356
Carcinomas	Ref			Ref		
Gonadal tumors	−14.29	−24.29—−4.29		−7.49	−19.68—4.69	
Lymphomas	4.89	−6.13—15.92		3.44	−7.50—14.38	
Other tumors^c^	−2.68	−13.40—8.04		1.99	−8.75—12.74	
Treatment^d^			**0.009**			**0.014**
Surgery	Ref			Ref		
Chemotherapy	16.27	5.76—26.79		15.62	5.03—26.22	
Radiotherapy and stem cell transplantation	12.44	2.50—22.38		7.31	−2.89—17.50	
Age at diagnosis			0.591			
21–30 years	Ref					
31–39 years	2.23	−5.98—10.44				
Time since diagnosis			0.152			
2–5 years	Ref					
6–13 years	5.83	−2.17—13.83				
Late effects			0.604			
No	Ref					
Yes	2.21	−6.19—10.61				
Relapse			0.325			
No	Ref					
Yes	6.89	−6.04—20.67				
Second cancer						
No Yes	Ref 5.36	−10.4—21.12	0.502			
CD-RISC 10						
Resilience sum score	0.28	−0.39—0.96	0.408			

*CI confidence interval, *Coeff*. coefficient, *Ref*. reference category

Bold font indicates statistically significant results

^a^ Educational achievement: low includes compulsory schooling and vocational training; high includes upper secondary education and university education

^b^ Employment status at study: Employed includes full-time and part-time employment. Other situations included homeworkers and those not employed

^c^ The category “other tumors” consists of leukemias, CNS neoplasms, sarcomas, nerve sheath tumors, and malignant melanoma

^d^ Treatment was coded hierarchically as “surgery only”; “chemotherapy (may have had surgery)”; radiotherapy and stem cell transplantation (may have had surgery and/or chemotherapy)”

^e^ P values for categorical independent variables with multiple levels were calculated using the Wald test

In our explorative analysis on the resilience items, the items *Adapt to change* (*p* = 0.035) and *See humorous side of things* (*p* = 0.029) were statistically significantly associated with the PTG sum score (Supplementary Information, Table S5). In our explorative analyses on the five PTG domains, results were similar as for the PTG sum score with some more assessed characteristics significantly associated with some of the PTG domains (Supplementary Information, Tables S3 to S7). Higher resilience was significantly associated with higher PTG in the domain *Personal strength*.

## Discussion

In our study, we found that Swiss YACS reported middle to high levels of PTG and resilience. Female survivors and those who had received chemotherapy reported higher PTG. Resilience was not associated with PTG.

(A)YAs with cancer are diagnosed during a unique period of their life, full of psychosocial changes. The moderate PTG levels of our YACS sample are comparable to a recent Swiss study, reporting moderate PTG levels in Swiss AYA cancer survivors [20]. A systematic review in AYA cancer patients reported generally moderate resilience levels, similar to ours [17]. The YACS in our sample were diagnosed during young adulthood (21 to 39 years of age) and on average aged 38 years (SD = 5 years) at time of assessment. Our results therefore add to the literature on positive outcomes in people being diagnosed in the AYA age range.

Female participants reported significantly higher PTG compared to males. There are mixed results in the literature regarding sex and the development of PTG [15, 20, 35, 36]. One possible explanation of our finding might be that women may be more likely than men to engage in emotional disclosure and receive social support, which have been linked to increased PTG [37]. Our exploratory analyses on the PTG domains showed this sex difference as well as the association with cancer treatment for some PTG domains as well. This pattern parallels findings in breast cancer survivors, where domain-level associations largely mirrored those for the global PTG score [38]. A similar distinction between broad, general effects and domain-specific effects on PTG has also been described in childhood cancer survivors [39].

In line with previous studies showing that receiving chemotherapy or experiencing unexpected complications during treatment was associated with higher levels of PTG, our study also found that YACS who underwent chemotherapy reported significantly higher levels of PTG than those treated with surgery only [35, 40, 41]. This finding aligns with the theoretical assumption that more threatening or life-disrupting experiences may trigger stronger PTG responses by encouraging deeper cognitive engagement and emotional processing [23]. Accordingly, the longer duration of chemotherapy may offer more opportunity for such processing, thereby facilitating PTG. Supporting this, Danhauer et al. (2015) found that breast cancer survivors who reported higher levels of PTG were more likely to have received chemotherapy compared to those with very low PTG [38]. Similarly, a recent study showed that breast cancer patients perceived chemotherapy as more emotionally burdensome and threatening than surgery, which may increase the potential for trauma-related reappraisal and growth [42].

There are differing findings on the relationship between resilience and PTG across different populations (e.g., cancer patients and survivors, persons after childhood abuse) [43, 44], and our results contribute to this complexity. Most studies found that there is a positive relationship between resilience and PTG, meaning that a more resilient individual also reported a higher level of PTG [27, 45]. For instance, a recent study among female patients with breast cancer found that resilience was only positively associated with PTG in patients who considered cancer as a traumatic experience [28]. As we did not assess perceived trauma, this may partly explain the flattened resilience–PTG association observed. It is conceivable, though speculative, that survivors who perceived their cancer as more traumatic might show a stronger resilience–PTG association. Another study identified a curvilinear relationship between resilience and PTG, whereby moderate levels of resilience were associated with the highest PTG levels, suggesting that low resilience may result in emotional overwhelm, while high resilience might limit the cognitive and emotional processing required for growth [46]. Furthermore, Seiler et al. (2019) suggested that the relationship between resilience and PTG may follow distinct temporal trajectories along the cancer continuum, with resilience playing a more prominent role during the acute treatment phase, while PTG tends to emerge later, particularly during long-term survivorship [47]. This may also help explain the lack of significant association found in our survivor sample, even though resilience as a whole was moderate to high, as were reported levels of PTG. The absence of an association at the global resilience level may also reflect limited statistical power in our sample. Another explanation may lie in the theoretical distinction between resilience and PTG. While resilience often reflects a return to baseline functioning after adversity, like a bounce back, PTG involves deeper cognitive transformation and meaning reconstruction through struggle [23, 47]. Even though they are both salutogenic constructs, this may lead to different pathways of adaptation [43, 48]. Additionally, a study including AYA cancer patients showed that resilience was positively correlated with quality of life and life satisfaction, but did not consistently predict the development of PTG [17].

Resilience is a multifaceted construct, indicating that some but not all components of resilience may be associated with PTG. In our exploratory analysis, the humor-related item “See humorous side of things” from the resilience scale was positively associated with PTG, despite its overall low endorsement in our YACS sample. This supports previous findings that cognitive reappraisal strategies, including humor, can promote PTG by facilitating emotional regulation, maintaining perspective, and fostering meaning-making [49].

The non-association of PTG and resilience in our study could reflect both theoretical and measurement complexities: standard resilience measures may not capture the dimensions associated with PTG, and the timing of assessment (cross-sectional versus longitudinal) may obscure dynamic relationships.

### Strengths and limitations

A major strength of our study among YACS is the use of internationally established and psychometrically validated instruments, the PTGI for posttraumatic growth and the CD-RISC 10 for resilience, ensuring both methodological rigor and comparability with international research. Moreover, the study benefited from collaboration with four different Swiss clinics, which facilitated participant recruitment.

Besides the strengths of the study, several limitations should be acknowledged. Firstly, the sample size was relatively small and limited to German-speaking participants, which may reduce the generalizability of findings for other language regions within Switzerland or other countries. Despite the similarity of our sample to the Swiss YACS population regarding gender and age at diagnosis, substantial differences in diagnosis, treatment, and second cancer history may affect the transferability of our results to the broader Swiss survivor population. Although the inclusion of four clinics facilitated recruitment, the number of participating institutions remained limited due to legal and organizational hurdles, which restricted broader outreach and may have introduced selection bias. Additionally, as the AYA age range spans distinct developmental stages [50], and about 60% of our study participants were 31 years or older at diagnosis, our findings may reflect this adulthood subgroup more strongly than AYAs being younger at diagnosis. Secondly, comparison between responders and non-responders was not possible, as former patients were contacted directly by the clinics, and data protection regulations prohibited us from accessing their identities prior to receiving written informed consent. This raises the possibility of selection bias, as we cannot rule out that survivors with less PTG were less inclined to participate, though other factors may also explain non-response. Notably, item-level missingness on the PTGI among respondents was low (n = 3 of 131). Thirdly, the cross-sectional design of our study limits conclusions regarding the directionality or causality of the observed associations. Relatedly, we modeled resilience as a predictor rather than a moderator of PTG, testing moderation would require a larger sample than available in our study. Fourthly, although the literature has established cancer as a potentially traumatic experience [14], our questionnaire did not assess whether participants subjectively perceived their own cancer as traumatic, which limits the interpretation of PTG scores. Finally, several analytical choices should be noted: categorization cut-offs followed previous publications rather than our sample's empirical distribution and our primary regression models focused on the PTG sum score, although we additionally conducted exploratory analyses examining the five PTGI domains; these exploratory item-level and domain-level analyses were not adjusted for multiple comparisons and should therefore be interpreted with caution. Our broader survey also covered topics such as family, friends, and psychosocial support, which were not analyzed here but may warrant dedicated attention in future work.

### Clinical implications

Based on our findings, several clinical implications emerge that may help inform supportive care practices for YACS.

- **Tailor psychosocial support to different adaptive pathways**: Our findings suggest that resilience and PTG represent distinct processes. Psychosocial programs should therefore differentiate between recovery-focused and growth-oriented support. For example, resilience training might focus on strengthening stable coping capacities, while PTG interventions could encourage deeper reflection and meaning-making [48].This distinction is supported by intervention research: randomized controlled trials of resilience-promoting interventions in cancer patients have primarily targeted the restoration and strengthening of coping resources [51], whereas meaning-centered group psychotherapy, focused on personal meaning-making, has shown specific effects on posttraumatic growth [52].
- **Recognize the individual variation in PTG experiences**: Given the wide range of PTG levels in our sample, psychosocial care should acknowledge that not all YACS experience the same levels of PTG. Some cancer patients or survivors may perceive growth, while others might feel pressured to report positive changes or experience illusory growth [53, 54]. Furthermore, our finding that female participants reported higher PTG than males highlights the need to consider gender-specific experiences and coping styles in post-cancer care. Thus, professionals should avoid assuming universal PTG and instead adopt a sensitive, individualized approach that validates both positive and negative post-cancer experiences.
- **Address the emotional impact of intensive treatments such as chemotherapy**: Survivorship care should provide space for reflection and guided processing for those who underwent intensive treatments, acknowledging both the psychological burden and potential for positive change.

Together, these implications emphasize the importance of nuanced, individualized psychosocial care that recognizes the diverse ways young adult cancer survivors respond to their experiences, whether through resilience, PTG, or both, and supports them accordingly across the survivorship trajectory. This may include subtle, low-threshold support formats (e.g., poster, flyer) to respect individual readiness and preferences.

## Conclusion

In our study, Swiss YACS reported middle to high levels of PTG and resilience. PTG was higher among females and those who received chemotherapy, while resilience showed no significant association with PTG. These findings support the hypothesis that PTG and resilience are distinct adaptation processes. While resilience may reflect returning to baseline functioning, PTG may involve deeper psychological change. Tailoring support to individual experiences can help YACS navigate the complex psychological aftermath of cancer more effectively.

## Supplementary Information

Below is the link to the electronic supplementary material.

Supplementary file1 (DOCX 274 KB)

## Data Availability

The data that support the findings of this study are available from the corresponding author upon reasonable request.

## Abbreviations

AYAs: Adolescent and young adults
CI: Confidence interval
CNS: Central nervous system
OR: Odds ratio
Ref: Reference category
N, n: Number
YACS: Young adult cancer survivors
