# Supplementary material for: Posttraumatic growth and resilience among survivors of cancer in young adulthood in Switzerland: a report from the Adolescent and Young Adult (AYA) Psychosocial Health Study
Source: Support Care Cancer. 2026 Nov 4;34(11):1192. doi: 10.1007/s00520-026-11383-6 (PMC13736483; doi:10.1007/s00520-026-11383-6)
Supplement: Supplementary file 1 [file 520_2026_11383_MOESM1_ESM.docx]

[Supportive Care in Cancer]

Appendix to the manuscript

**Title: Posttraumatic growth and resilience among survivors of cancer in young adulthood in Switzerland: a report from the Adolescent and Young Adult (AYA) Psychosocial Health Study**

Céline Bolliger^1^, Luzius Mader^2^, Mohsen Mousavi^3^, Beat Müller^4^, Marcus Vetter^5^, Katharina Roser^1*^

^1^ Faculty of Health Sciences and Medicine, University of Lucerne, Lucerne, Switzerland

^2^ Cancer Registry Bern and Solothurn, Bern, Switzerland

^3^ Cancer Registry of Eastern Switzerland, St.Gallen, Switzerland

^4^ Department for Medical Oncology, Cantonal Hospital of Lucerne, Lucerne, Switzerland

^5^ Center for Oncology and Hematology, Cancer Center Baselland, Cantonal Hospital Baselland Liestal, Switzerland

* Correspondence: [katharina.roser@unilu.ch](mailto:katharina.roser@unilu.ch); Tel.: +41 41 229 59 56

**Overview**

Supplementary Information 1 – Figure S1: Study flow chart

Supplementary Information 2 – Table S1: Comparison of YACS participants and the Swiss 2-year YACS population

Supplementary Information 3 – Figure S2: Proportion of participants endorsing the PTG items

Supplementary Information 4 – Figure S3: Proportion of participants endorsing the resilience items

Supplementary Information 5 – Table S2: Explorative analysis of the resilience items in relation to PTG

Supplementary Information 6 – Table S3: Explorative univariable and multivariable linear regressions of the different characteristics in relation to the PTG domain *Relating to others*

Supplementary Information 6 – Table S4: Explorative univariable and multivariable linear regressions of the different characteristics in relation to the PTG domain *New possibilities*

Supplementary Information 6 – Table S5: Explorative univariable and multivariable linear regressions of the different characteristics in relation to the PTG domain *Personal strength*

Supplementary Information 6 – Table S6: Explorative univariable and multivariable linear regressions of the different characteristics in relation to the PTG domain *Spiritual change*

Supplementary Information 6 – Table S7: Explorative univariable and multivariable linear regressions of the different characteristics in relation to the PTG domain *Appreciation of life*

**Supplementary Information 1 – Figure S1: Study flow chart**

**Contacted survivors of young adult cancer**

n = 678 (100%)

**No response**

n = 442 (65.2%)

**Contacted**

**Returned** (e.g. wrong address, deceased)

n = 50 (7.4%)

**Refusal to participate**

n = 11 (1.6%)

**Responses**

n = 236 (34.8%)

**Missing informed consent**

n=27 (4%)

**Missing questionnaire**

n= 7 (1%)

**Did not meet inclusion criteria** (e.g., not diagnosed two years ago, did not live in Switzerland)

n=7 (1.2%)

**No data on PTG and resilience**

n= 3 (0.6%)

**Positive responses**

n = 175 (25.8%)

**Response**

**Study participants included survivors of young adult cancer**

n = 131 (19.3%)

**Included**

**Figure S1.** Study flow chart: recruitment of study participants.

**Supplementary Information 2 – Table S1: Comparison of YACS participants and the Swiss 2-year YACS population**

**Table S1.** Comparison of the YACS population of the Psychosocial health study and the Swiss 2-year YACS population.

|  | **Young adult cancer survivors (YACS)** | | | | | | |
| --- | --- | --- | --- | --- | --- | --- | --- |
|  | **YACS Population of the Psychosocial Health Study^a^** | | **YACS 2-year Population of Switzerland**^b^ | | | | **Chi^2^ test** |
|  | **Observed**  N=131 | | **Observed**^c^  N=13’290 | | **Estimated**^d^  N=15’603.3 | |  |
|  | n | % | n | % | n | % | **p-value** |
| Sex  Male  Female | 50  81 | 38.2  61.8 | 5’704 7’586 | 42.9  57.1 | 6708.1  8895.1 | 43.0  57.0 | 0.267 |
| Age at diagnosis  20-24^a^  25-29  30-34  35-39 | 16  27  49  39 | 12.2  20.6  37.4  29.7 | 1’245  2’504  4’011  5’530 | 9.4  18.8  30.2  41.6 | 1’476.2  2’948.6  4’692.4  6’486.0 | 9.5  18.930.141.6 | 0.048 |
| Diagnosis (according to Barr et al. 2020)   1. Leukemias and related disorders 2. Lymphomas 3. CNS and other intracranial/intraspinal neoplasms 4. Sarcomas 5. Blood and lymphatic vessel tumor^e^ 6. Nerve sheath tumors 7. Gonadal and related tumors 8. Melanoma, malignant 9. Carcinomas 10. Miscellaneous specified neoplasms^e^ 11. Unspecified malignant neoplasms except CNS^e^ | 5  23  6  3  -  1  31  10  52  -  - | 3.8  17.5  4.6  2.3  -  0.8  23.6  7.6  39.7  -  - | 541  1’412  1’121  440  79  153  2’605  1’923  5’500  24  32 | 4.1  10.6  8.4  3.3  0.6  1.2  15.5  14.5  41.4  0.2  0.2 | 633.9  1’651.7  1’326.4  516.7  92.2  1’81.3  2’443.4  2’248.7  6’443.0  28.2  37.8 | 4.1  10.6  8.5  3.3  0.6  1.2  15.7  14.4  41.3  0.2  0.2 | **0.011** |
| Treatment^f^  Surgery only  Chemotherapy  Radiotherapy  Stem cell transplantation | 30  43  49  9 | 22.9  32.8  37.4  6.9 | 5’277  2’101  2’832  101 | 39.7  15.8  21.3  0.8 | 6’253  2’491  3’326  118 | 40.1  16.0  21.3  0.8 | **p<0.001** |
| Second cancer^d^  No  Yes | 122  9 | 93.1  6.9 | 12’876  202 | 98.5  1.5 | 15129.3  231.3 | 98.5  1.5 | **p<0.001** |

Abbreviations: YACS, Young adult cancer survivor; CNS, Central nervous system.

Notes: We conducted Chi-square tests with the estimated number of cancer cases in the YA population of Switzerland. Bold font indicates statistically significant results (p<0.05).

^a^ The YA population from the psychosocial health study includes individuals diagnosed between the ages of 21 to 39 years (inclusion criteria)

^b^ The YA population of Switzerland includes individuals diagnosed with cancer between the ages of 20-39 years, and the initial cancer diagnosis was between 1980 and 2019. Data covers the whole of Switzerland, and participants were registered in the Swiss Childhood Cancer Registry (ChCR, age 15-19 years) and at the National Agency for Cancer Registration (NACR, age 20-39 years), respectively. Furthermore, the YA population was restricted to at least 2 years after diagnosis.

^c^ Observed: number of cancer cases collected by the ChCR and/or the NACR. Covered 63% of the AYA cancers diagnosed in Switzerland between 1980-2019.

^d^ Estimated: number of cases after extrapolation of the observed cancer cases to the whole of Switzerland to acquire 100% coverage. Characteristic-specific totals don’t necessarily add up to the same estimated column total.

^e^ Cancer cases in these three cancer categories from Barr et al. 2020 – (5) Blood and lymphatic vessel tumors, (10) Miscellaneous specified neoplasms, and (11) Unspecified malignant neoplasms except CNS –have been recorded under different categories.

^f^ Hierarchically coded: chemotherapy may include surgery, radiotherapy may include surgery and/or chemotherapy, stem cell transplantation may include surgery and/or chemotherapy and/or radiotherapy. Those don’t add up to the column total for the populations of the epidemiological study, because of cancer cases that received other treatments or had unknown treatment.

**Supplementary Information 3 – Figure S2: Proportion of participants endorsing the PTG items**

**Figure S2.** Proportion of participants endorsing the posttraumatic growth (PTG) items measured by the Posttraumatic Growth Inventory (PTGI) at low (0,1), middle (2,3) or high (4,5) level. Each PTGI item is rated using a 6-point Likert scale (0=not at all, 1=barley, 2=a little, 3 =moderately, 4=very much, 5=extremely).

**Supplementary Information 4 – Figure S3: Proportion of participants endorsing the resilience items**

**Figure S3.** Proportion of participants endorsing the resilience items measured by the CD-RISC 10 at low (0,1), middle (2), or high (3,4) level. Each CD-RISC 10 item is rated using a 5-point Likert scale (0=not at all, 1=rarely true, 2=sometimes true, 3=often true, 4=almost always true).

**Supplementary Information 5 – Table S2: Explorative analysis of the resilience items in relation to PTG**

**Table S2:** Explorative analysis investigating the associations of the resilience items with the PTG sum score.

|  | Univariable linear regression | | |
| --- | --- | --- | --- |
|  | Coeff | 95% CI | p value |
| **CD-RISC 10** |  |  |  |
| Adapt to change | 5.60 | 0.40 - 10.78 | **0.035** |
| Can deal with whatever comes | 2.22 | -2.63 - 7.07 | 0.367 |
| See humorous side of things | 4.59 | 0.48 - 8.70 | **0.029** |
| Coping with stress strengthens | 0.23 | -3.78 - 4.23 | 0.911 |
| Tend to bounce back after illness or hardship | 1.86 | -2.86 - 6.60 | 0.436 |
| You can achieve your goals | 0.48 | -5.02 - 5.98 | 0.863 |
| Under pressure, focus and think clearly | -1.36 | -5.51 - 2.77 | 0.515 |
| Not easily discouraged by failure | -0.04 | -3.98 - 3.90 | 0.983 |
| Think of self as strong person | 2.30 | -1.94 - 6.54 | 0.285 |
| Can handle unpleasant feelings | -1.65 | -5.79 - 2.49 | 0.433 |

Abbreviations: CI, confidence interval; Coeff, coefficient.

Note: Bold font indicates statistically significant results (p<0.05).

**Supplementary Information 6 – Table S3: Explorative univariable and multivariable linear regressions of the different characteristics in relation to the PTG domain *Relating to others***

**Table S3.** Univariable and multivariable linear regressions investigating the associations of sociodemographic, cancer-related characteristics and resilience with the PTG domain *Relating to others*.

|  | Univariable linear regression | | | Multivariable linear regression | | |
| --- | --- | --- | --- | --- | --- | --- |
|  | Coeff | 95% CI | p value | Coeff | 95% CI | p value^e^ |
| Sociodemographic characteristics | | | | | | |
| **Sex** |  |  | **0.007** |  |  | **0.128** |
| Male | Ref. |  |  | Ref. |  |  |
| Female | 0.55 | 0.15 – 0.95 |  | 0.37 | -0.11 – 0.74 |  |
| **Age at study** |  |  | 0.759 |  |  |  |
| 21-30 years | Ref. |  |  |  |  |  |
| >=31 | 0.10 | -0.56 – 0.77 |  |  |  |  |
| **Migration background** |  |  | **0.054** |  |  | **0.011** |
| No | Ref. |  |  | Ref. |  |  |
| Yes | -1.00 | -2.02 – 0.02 |  | -1.29 | -2.27 - -0.30 |  |
| **Educational achievement**^a^ |  |  | 0.719 |  |  |  |
| Low | Ref. |  |  |  |  |  |
| High | -0.07 | -0.48 -0.33 |  |  |  |  |
| **Employment status**^b^ |  |  | 0.719 |  |  |  |
| Employed | Ref. |  |  |  |  |  |
| Other situation | 0.08 | -0.35 – 0.51 |  |  |  |  |
| **Partnership** |  |  | **0.034** |  |  | **0.044** |
| No | Ref. |  |  | Ref. |  |  |
| Yes | -0.51 | -0.98 - -0.04 |  | -0.46 | -0.91 - -0.01 |  |
| Cancer-related characteristics |  |  |  |  |  |  |
| **Diagnosis** |  |  | **0.037** |  |  | 0.465 |
| Carcinomas | Ref. |  |  | Ref. |  |  |
| Gonadal tumors | -0.63 | -1.13 - -0.12 |  | -0.43 | -1.04 – 1.18 |  |
| Lymphomas | 0.19 | -0.36 – 0.75 |  | 0.06 | -0.48 – 0.61 |  |
| Other tumors^c^ | -0.08 | -0.62 – 0.46 |  | -0.05 | -0.59 – 0.49 |  |
| **Treatment**^d^ |  |  | **0.053** |  |  | 0.078 |
| Surgery | Ref. |  |  | Ref. |  |  |
| Chemotherapy | 0.59 | 0.06 – 1.12 |  | 0.61 | 0.08 – 1.14 |  |
| Radiotherapy and stem cell transplantation | 0.56 | 0.06 – 1.06 |  | 0.37 | -0.14 – 0.88 |  |
| **Age at diagnosis** |  |  | 0.368 |  |  |  |
| 21-30 years | Ref. |  |  |  |  |  |
| 31-39 years | 0.19 | -0.22 – 0.59 |  |  |  |  |
| **Time since diagnosis** |  |  | 0.197 |  |  |  |
| 2-5 years | Ref. |  |  |  |  |  |
| 6-13 years | 0.26 | -0.14 – 0.66 |  |  |  |  |
| **Late effects** |  |  | 0.401 |  |  |  |
| No | Ref. |  |  |  |  |  |
| Yes | 0.18 | -0.24 – 0.59 |  |  |  |  |
| **Relapse** |  |  | 0.611 |  |  |  |
| No | Ref. |  |  |  |  |  |
| Yes | 0.18 | -0.51 – 0.86 |  |  |  |  |
| **Second cancer** |  |  | 0.851 |  |  |  |
| No | Ref. |  |  |  |  |  |
| Yes | 0.07 | -0.71- 0.86 |  |  |  |  |
| **CD-RISC 10** |  |  |  |  |  |  |
| Resilience sum score | 0.00 | -0.03 – 0.04 | 0.808 |  |  |  |

Abbreviations: CI, confidence interval; Coeff, coefficient; Ref, reference category.

Note: Bold font indicates statistically significant results.

^a^ Educational achievement: low includes compulsory schooling and vocational training; high includes upper secondary education and university education

^b^ Employment status at study: Employed includes full-time and part-time employment. Other situations included homeworkers and those not employed.

^c^ The category “other tumors” consists of leukemias, CNS neoplasms, sarcomas, nerve sheath tumors, and malignant melanoma.

^d^ Treatment was coded hierarchically as “surgery only”; “chemotherapy (may have had surgery)”; radiotherapy and stem cell transplantation (may have had surgery and/or chemotherapy)”.

^e^ P values for categorical independent variables with multiple levels were calculated using the Wald test.

**Supplementary Information 7 – Table S4: Explorative univariable and multivariable linear regressions of the different characteristics in relation to the PTG domain *New possibilities***

**Table S3.** Univariable and multivariable linear regressions investigating the associations of sociodemographic, cancer-related characteristics and resilience with the PTG domain *New possibilities*.

|  | Univariable linear regression | | | Multivariable linear regression | | |
| --- | --- | --- | --- | --- | --- | --- |
|  | Coeff | 95% CI | p value | Coeff | 95% CI | p value^e^ |
| Sociodemographic characteristics | | | | | | |
| **Sex** |  |  | **0.004** |  |  | 0.099 |
| Male | Ref. |  |  | Ref. |  |  |
| Female | 0.67 | 0.22-1.12 |  | 0.45 | -0.09 – 0.98 |  |
| **Age at study** |  |  | 0.528 |  |  |  |
| 21-30 years | Ref. |  |  |  |  |  |
| >=31 | 0.24 | -0.51 – 0.99 |  |  |  |  |
| **Migration background** |  |  | 0.896 |  |  |  |
| No | Ref. |  |  |  |  |  |
| Yes | -0.08 | -1.25 – 1.09 |  |  |  |  |
| **Educational achievement**^a^ |  |  | 0.880 |  |  |  |
| Low | Ref. |  |  |  |  |  |
| High | -0.04 | -0.50 – 0.43 |  |  |  |  |
| **Employment status**^b^ |  |  | 0.618 |  |  |  |
| Employed | Ref. |  |  |  |  |  |
| Other situation | 0.12 | -0.37 – 0.61 |  |  |  |  |
| **Partnership** |  |  | 0.590 |  |  |  |
| No | Ref. |  |  |  |  |  |
| Yes | -0.15 | -0.69 – 0.39 |  |  |  |  |
| Cancer-related characteristics |  |  |  |  |  |  |
| **Diagnosis** |  |  | **0.018** |  |  | 0.411 |
| Carcinomas | Ref. |  |  | Ref. |  |  |
| Gonadal tumors | -0.79 | -1.35 - -0.23 |  | -0.50 | -1.19 – 1.18 |  |
| Lymphomas | 0.19 | -0.43 – 0.81 |  | 0.06 | -0.56 – 0.67 |  |
| Other tumors^c^ | -0.26 | -0.87 – 0.34 |  | 0.00 | -0.61 – 0.60 |  |
| **Treatment**^d^ |  |  | **0.002** |  |  | **0.003** |
| Surgery | Ref. |  |  | Ref. |  |  |
| Chemotherapy | 1.05 | 0.47 – 1.63 |  | 1.02 | 0.43 – 1.61 |  |
| Radiotherapy and stem cell transplantation | 0.74 | 0.19 – 1.30 |  | 0.47 | -0.10 – 1.05 |  |
| **Age at diagnosis** |  | - | 0.687 |  |  |  |
| 21-30 years | Ref. |  |  |  |  |  |
| 31-39 years | 0.09 | -0.37 – 0.56 |  |  |  |  |
| **Time since diagnosis** |  |  | 0.511 |  |  |  |
| 2-5 years | Ref. |  |  |  |  |  |
| 6-13 years | 0.15 | -0.30 – 0.60 |  |  |  |  |
| **Late effects** |  |  | 0.685 |  |  |  |
| No | Ref. |  |  |  |  |  |
| Yes | 0.10 | -0.37 – 0.57 |  |  |  |  |
| **Relapse** |  |  | 0.179 |  |  |  |
| No | Ref. |  |  |  |  |  |
| Yes | 0.53 | -0.24 – 1.30 |  |  |  |  |
| **Second cancer** |  |  | 0.593 |  |  |  |
| No | Ref. |  |  |  |  |  |
| Yes | 0.24 | -0.65 – 1.13 |  |  |  |  |
| **CD-RISC 10** |  |  |  |  |  |  |
| Resilience sum score | 0.01 | -0.02 – 0.05 | 0.465 |  |  |  |

Abbreviations: CI, confidence interval; Coeff, coefficient; Ref, reference category.

Note: Bold font indicates statistically significant results.

^a^ Educational achievement: low includes compulsory schooling and vocational training; high includes upper secondary education and university education

^b^ Employment status at study: Employed includes full-time and part-time employment. Other situations included homeworkers and those not employed.

^c^ The category “other tumors” consists of leukemias, CNS neoplasms, sarcomas, nerve sheath tumors, and malignant melanoma.

^d^ Treatment was coded hierarchically as “surgery only”; “chemotherapy (may have had surgery)”; radiotherapy and stem cell transplantation (may have had surgery and/or chemotherapy)”.

^e^ P values for categorical independent variables with multiple levels were calculated using the Wald test.

**Supplementary Information 8 – Table S5: Explorative univariable and multivariable linear regressions of the different characteristics in relation to the PTG domain *Personal strength***

**Table S3.** Univariable and multivariable linear regressions investigating the associations of sociodemographic, cancer-related characteristics and resilience with the PTG domain *Personal strength*.

|  | Univariable linear regression | | | Multivariable linear regression | | |
| --- | --- | --- | --- | --- | --- | --- |
|  | Coeff | 95% CI | p value | Coeff | 95% CI | p value^e^ |
| Sociodemographic characteristics | | | | | | |
| **Sex** |  |  | **0.002** |  |  | **0.014** |
| Male | Ref. |  |  | Ref. |  |  |
| Female | 0.72 | 0.27 – 1.16 |  | 0.66 | 0.14 – 1.18 |  |
| **Age at study** |  |  | 0.832 |  |  |  |
| 21-30 years | Ref. |  |  |  |  |  |
| >=31 | 0.08 | -0.68 – 0.84 |  |  |  |  |
| **Migration background** |  |  | 0.837 |  |  |  |
| No | Ref. |  |  |  |  |  |
| Yes | 0.12 | -1.06 – 1.31 |  |  |  |  |
| **Educational achievement**^a^ |  |  | 0.719 |  |  |  |
| Low | Ref. |  |  |  |  |  |
| High | 0.09 | -0.38 – 0.55 |  |  |  |  |
| **Employment status**^b^ |  |  | 0.524 |  |  |  |
| Employed | Ref. |  |  |  |  |  |
| Other situation | 0.16 | -0.34 – 0.66 |  |  |  |  |
| **Partnership** |  |  | **0.072** |  |  | 0.094 |
| No | Ref. |  |  | Ref. |  |  |
| Yes | -0.50 | -1.04 – 0.04 |  | -0.43 | -0.93 – 0.07 |  |
| Cancer-related characteristics |  |  |  |  |  |  |
| **Diagnosis** |  |  | **0.005** |  |  | 0.094 |
| Carcinomas | Ref. |  |  | Ref. |  |  |
| Gonadal tumors | -0.73 | -1.30 - -0.17 |  | -0.37 | -1.03 – 0.30 |  |
| Lymphomas | 0.48 | -0.15 – 1.10 |  | 0.30 | -0.31 – 0.91 |  |
| Other tumors^c^ | 0.12 | -0.48 – 0.73 |  | 0.43 | -0.17 – 1.03 |  |
| **Treatment**^d^ |  |  | **0.083** |  |  | **0.042** |
| Surgery | Ref. |  |  | Ref. |  |  |
| Chemotherapy | 0.66 | 0.05 – 1.27 |  | 0.12 | 0.14 – 1.30 |  |
| Radiotherapy and stem cell transplantation | 0.55 | -0.03 – 1.12 |  | 0.28 | -0.28 – 0.84 |  |
| **Age at diagnosis** |  | - | 0.727 |  |  |  |
| 21-30 years | Ref. |  |  |  |  |  |
| 31-39 years | 0.08 | -0.39 – 0.55 |  |  |  |  |
| **Time since diagnosis** |  |  | 0.182 |  |  |  |
| 2-5 years | Ref. |  |  |  |  |  |
| 6-13 years | 0.31 | -0.15 – 0.77 |  |  |  |  |
| **Late effects** |  |  | 0.845 |  |  |  |
| No | Ref. |  |  |  |  |  |
| Yes | -0.05 | -0.53 – 0.43 |  |  |  |  |
| **Relapse** |  |  | 0.143 |  |  |  |
| No | Ref. |  |  |  |  |  |
| Yes | 0.58 | -0.20 – 1.36 |  |  |  |  |
| **Second cancer** |  |  | 0.238 |  |  |  |
| No | Ref. |  |  |  |  |  |
| Yes | 0.54 | -0.36 – 1.43 |  |  |  |  |
| **CD-RISC 10** |  |  |  |  |  |  |
| Resilience sum score | 0.04 | 0.01 – 0.08 | **0.023** | 0.06 | 0.02 – 0.10 | **0.001** |

Abbreviations: CI, confidence interval; Coeff, coefficient; Ref, reference category.

Note: Bold font indicates statistically significant results.

^a^ Educational achievement: low includes compulsory schooling and vocational training; high includes upper secondary education and university education

^b^ Employment status at study: Employed includes full-time and part-time employment. Other situations included homeworkers and those not employed.

^c^ The category “other tumors” consists of leukemias, CNS neoplasms, sarcomas, nerve sheath tumors, and malignant melanoma.

^d^ Treatment was coded hierarchically as “surgery only”; “chemotherapy (may have had surgery)”; radiotherapy and stem cell transplantation (may have had surgery and/or chemotherapy)”.

^e^ P values for categorical independent variables with multiple levels were calculated using the Wald test.

**Supplementary Information 9 – Table S6: Explorative univariable and multivariable linear regressions of the different characteristics in relation to the PTG domain *Spiritual change***

**Table S3.** Univariable and multivariable linear regressions investigating the associations of sociodemographic, cancer-related characteristics and resilience with the PTG domain *Spiritual change*.

|  | Univariable linear regression | | | Multivariable linear regression | | |
| --- | --- | --- | --- | --- | --- | --- |
|  | Coeff | 95% CI | p value | Coeff | 95% CI | p value^e^ |
| Sociodemographic characteristics | | | | | | |
| **Sex** |  |  | **0.004** |  |  | **<0.001** |
| Male | Ref. |  |  | Ref. |  |  |
| Female | 0.72 | 0.23 – 1.21 |  | 0.88 | 0.40 – 1.37 |  |
| **Age at study** |  |  | 0.195 |  |  |  |
| 21-30 years | Ref. |  |  |  |  |  |
| >=31 | -0.53 | -1.35 – 0.28 |  |  |  |  |
| **Migration background** |  |  | 0.076 |  |  |  |
| No | Ref. |  |  |  |  |  |
| Yes | -1.14 | -2.40 – 0.12 |  |  |  |  |
| **Educational achievement**^a^ |  |  | 0.872 |  |  |  |
| Low | Ref. |  |  |  |  |  |
| High | 0.04 | -0.46 -0.54 |  |  |  |  |
| **Employment status**^b^ |  |  | 0.610 |  |  |  |
| Employed | Ref. |  |  |  |  |  |
| Other situation | 0.14 | -0.40 – 0.67 |  |  |  |  |
| **Partnership** |  |  | 0.855 |  |  |  |
| No | Ref. |  |  |  |  |  |
| Yes | -0.05 | -0.64 – 0.53 |  |  |  |  |
| Cancer-related characteristics |  |  |  |  |  |  |
| **Diagnosis** |  |  | 0.414 |  |  |  |
| Carcinomas | Ref. |  |  |  |  |  |
| Gonadal tumors | -0.37 | -1.00 – 0.26 |  |  |  |  |
| Lymphomas | 0.22 | -0.47 – 0.92 |  |  |  |  |
| Other tumors^c^ | 0.34 | -0.93 – 0.43 |  |  |  |  |
| **Treatment**^d^ |  |  | **0.014** |  |  | **0.001** |
| Surgery | Ref. |  |  | Ref. |  |  |
| Chemotherapy | 0.79 | 0.14 – 1.43 |  | 0.78 | 0.16 – 1.40 |  |
| Radiotherapy and stem cell transplantation | 0.05 | -0.57 – 0.66 |  | -0.18 | -0.78 – 0.41 |  |
| **Age at diagnosis** |  | - | 0.822 |  |  |  |
| 21-30 years | Ref. |  |  |  |  |  |
| 31-39 years | 0.06 | -0.45 – 0.56 |  |  |  |  |
| **Time since diagnosis** |  |  | 0.175 |  |  |  |
| 2-5 years | Ref. |  |  |  |  |  |
| 6-13 years | 0.34 | -0.15 – 0.83 |  |  |  |  |
| **Late effects** |  |  | 0.383 |  |  |  |
| No | Ref. |  |  |  |  |  |
| Yes | 0.23 | -0.29 -0.74 |  |  |  |  |
| **Relapse** |  |  | 0.063 |  |  |  |
| No | Ref. |  |  |  |  |  |
| Yes | -0.79 | -1.63 – 0.43 |  |  |  |  |
| **Second cancer** |  |  | 0.650 |  |  |  |
| No | Ref. |  |  |  |  |  |
| Yes | -0.22 | -1.19 – 0.74 |  |  |  |  |
| **CD-RISC 10** |  |  |  |  |  |  |
| Resilience sum score | -0.02 | -0.06 – 0.02 | 0.293 |  |  |  |

Abbreviations: CI, confidence interval; Coeff, coefficient; Ref, reference category.

Note: Bold font indicates statistically significant results.

^a^ Educational achievement: low includes compulsory schooling and vocational training; high includes upper secondary education and university education

^b^ Employment status at study: Employed includes full-time and part-time employment. Other situations included homeworkers and those not employed.

^c^ The category “other tumors” consists of leukemias, CNS neoplasms, sarcomas, nerve sheath tumors, and malignant melanoma.

^d^ Treatment was coded hierarchically as “surgery only”; “chemotherapy (may have had surgery)”; radiotherapy and stem cell transplantation (may have had surgery and/or chemotherapy)”.

^e^ P values for categorical independent variables with multiple levels were calculated using the Wald test.

**Supplementary Information 10 – Table S7: Explorative univariable and multivariable linear regressions of the different characteristics in relation to the PTG domain *Appreciation of life***

**Table S3.** Univariable and multivariable linear regressions investigating the associations of sociodemographic, cancer-related characteristics and resilience with the PTG domain *Appreciation of life*.

|  | Univariable linear regression | | | Multivariable linear regression | | |
| --- | --- | --- | --- | --- | --- | --- |
|  | Coeff | 95% CI | p value | Coeff | 95% CI | p value^e^ |
| Sociodemographic characteristics | | | | | | |
| **Sex** |  |  | **0.003** |  |  | 0.078 |
| Male | Ref. |  |  | Ref. |  |  |
| Female | 0.70 | 0.25 – 1.15 |  | 0.49 | -0.06 – 1.04 |  |
| **Age at study** |  |  | 0.393 |  |  |  |
| 21-30 years | Ref. |  |  |  |  |  |
| >=31 | 0.33 | -0.43 – 1.08 |  |  |  |  |
| **Migration background** |  |  | 0.996 |  |  |  |
| No | Ref. |  |  |  |  |  |
| Yes | 0.00 | -1.18 – 1.18 |  |  |  |  |
| **Educational achievement**^a^ |  |  | 0.983 |  |  |  |
| Low | Ref. |  |  |  |  |  |
| High | 0.00 | -0.47 – 0.46 |  |  |  |  |
| **Employment status**^b^ |  |  | 0.479 |  |  |  |
| Employed | Ref. |  |  |  |  |  |
| Other situation | 0.18 | -0.32 – 0.67 |  |  |  |  |
| **Partnership** |  |  | 0.612 |  |  |  |
| No | Ref. |  |  |  |  |  |
| Yes | -0.14 | -0.68 – 0.40 |  |  |  |  |
| Cancer-related characteristics |  |  |  |  |  |  |
| **Diagnosis** |  |  | **0.040** |  |  | 0.735 |
| Carcinomas | Ref. |  |  | Ref. |  |  |
| Gonadal tumors | -0.77 | -1.34 - -0.19 |  | -0.35 | -1.06 – 0.35 |  |
| Lymphomas | 0.08 | -0.55 - 0.71 |  | 0.00 | -0.63 – 0.63 |  |
| Other tumors^c^ | -0.27 | -0.88 – 0.35 |  | 0.01 | -0.61 – 0.62 |  |
| **Treatment**^d^ |  |  | **0.005** |  |  | **0.019** |
| Surgery | Ref. |  |  | Ref. |  |  |
| Chemotherapy | 0.89 | 0.29 – 1.48 |  | 0.88 | 0.27 – 1.49 |  |
| Radiotherapy and stem cell transplantation | 0.84 | 0.28 – 1.40 |  | 0.62 | 0.03 – 1.20 |  |
| **Age at diagnosis** |  |  | 0.980 |  |  |  |
| 21-30 years | Ref. |  |  |  |  |  |
| 31-39 years | 0.01 | -0.46 – 0.47 |  |  |  |  |
| **Time since diagnosis** |  |  | 0.053 |  |  |  |
| 2-5 years | Ref. |  |  |  |  |  |
| 6-13 years | 0.44 | -0.01 – 0.89 |  |  |  |  |
| **Late effects** |  |  |  |  |  |  |
| No | Ref. |  |  |  |  |  |
| Yes | 0.07 | -0.40 – 0.55 |  |  |  |  |
| **Relapse** |  |  | 0.055 |  |  |  |
| No | Ref. |  |  |  |  |  |
| Yes | 0.76 | -0.02 – 1.53 |  |  |  |  |
| **Second cancer** |  |  | 0.151 |  |  |  |
| No | Ref. |  |  |  |  |  |
| Yes | 0.65 | -0.24 – 1.53 |  |  |  |  |
| **CD-RISC 10** |  |  |  |  |  |  |
| Resilience sum score | 0.02 | -0.02 – 0.06 | 0.309 |  |  |  |

Abbreviations: CI, confidence interval; Coeff, coefficient; Ref, reference category.

Note: Bold font indicates statistically significant results.

^a^ Educational achievement: low includes compulsory schooling and vocational training; high includes upper secondary education and university education

^b^ Employment status at study: Employed includes full-time and part-time employment. Other situations included homeworkers and those not employed.

^c^ The category “other tumors” consists of leukemias, CNS neoplasms, sarcomas, nerve sheath tumors, and malignant melanoma.

^d^ Treatment was coded hierarchically as “surgery only”; “chemotherapy (may have had surgery)”; radiotherapy and stem cell transplantation (may have had surgery and/or chemotherapy)”.

^e^ P values for categorical independent variables with multiple levels were calculated using the Wald test.
